# Supplementary material for: The effects of genital myiasis on the diversity of the vaginal microbiota in female Bactrian camels
Source: BMC Vet Res. 2022 Mar 5;18:87. doi: 10.1186/s12917-022-03189-5 (PMC8897907; doi:10.1186/s12917-022-03189-5)
Supplement: Supplementary file 5 — Additional file 5. [file 12917_2022_3189_MOESM5_ESM.zip › MPL201709200_16s_yy/Treat1/css/js_css/jsPDF-master.zip!/jsPDF-master/docs/libs_require_require.js.html]

libs/require/require.js - Documentation


## Home

### Classes

- FontObject
- jsPDF
- PubSub

### Global

- addFont
- addMetadata
- addPage
- CapJoinStyles
- circle
- CssColors
- ellipse
- getFontList
- html2pdf
- http
- lines
- lstext
- output
- rect
- requirejs
- reset
- roundedRect
- save
- setDisplayMode
- setDrawColor
- setFillColor
- setFont
- setFontSize
- setFontStyle
- setLineCap
- setLineJoin
- setLineWidth
- setPage
- setProperties
- setTextColor
- text
- toUrl
- triangle
- triggerEvent

# libs/require/require.js

```
/** vim: et:ts=4:sw=4:sts=4
 * @license RequireJS 2.1.15 Copyright (c) 2010-2014, The Dojo Foundation All Rights Reserved.
 * Available via the MIT or new BSD license.
 * see: http://github.com/jrburke/requirejs for details
 */
//Not using strict: uneven strict support in browsers, #392, and causes
//problems with requirejs.exec()/transpiler plugins that may not be strict.
/*jslint regexp: true, nomen: true, sloppy: true */
/*global window, navigator, document, importScripts, setTimeout, opera */

var requirejs, require, define;
(function(global) {
	var req, s, head, baseElement, dataMain, src, interactiveScript, currentlyAddingScript, mainScript, subPath, version = '2.1.15', commentRegExp = /(\/\*([\s\S]*?)\*\/|([^:]|^)\/\/(.*)$)/mg, cjsRequireRegExp = /[^.]\s*require\s*\(\s*["']([^'"\s]+)["']\s*\)/g, jsSuffixRegExp = /\.js$/, currDirRegExp = /^\.\//, op = Object.prototype, ostring = op.toString, hasOwn = op.hasOwnProperty, ap = Array.prototype, apsp = ap.splice, isBrowser = !!(typeof window !== 'undefined'
			&& typeof navigator !== 'undefined' && window.document), isWebWorker = !isBrowser && typeof importScripts !== 'undefined',
	//PS3 indicates loaded and complete, but need to wait for complete
	//specifically. Sequence is 'loading', 'loaded', execution,
	// then 'complete'. The UA check is unfortunate, but not sure how
	//to feature test w/o causing perf issues.
	readyRegExp = isBrowser && navigator.platform === 'PLAYSTATION 3' ? /^complete$/ : /^(complete|loaded)$/, defContextName = '_',
	//Oh the tragedy, detecting opera. See the usage of isOpera for reason.
	isOpera = typeof opera !== 'undefined' && opera.toString() === '[object Opera]', contexts = {}, cfg = {}, globalDefQueue = [], useInteractive = false;

	function isFunction(it) {
		return ostring.call(it) === '[object Function]';
	}

	function isArray(it) {
		return ostring.call(it) === '[object Array]';
	}

	/**
	 * Helper function for iterating over an array. If the func returns
	 * a true value, it will break out of the loop.
	 */
	function each(ary,func) {
		if (ary) {
			var i;
			for (i = 0; i < ary.length; i += 1) {
				if (ary[i] && func(ary[i], i, ary)) {
					break;
				}
			}
		}
	}

	/**
	 * Helper function for iterating over an array backwards. If the func
	 * returns a true value, it will break out of the loop.
	 */
	function eachReverse(ary,func) {
		if (ary) {
			var i;
			for (i = ary.length - 1; i > -1; i -= 1) {
				if (ary[i] && func(ary[i], i, ary)) {
					break;
				}
			}
		}
	}

	function hasProp(obj,prop) {
		return hasOwn.call(obj, prop);
	}

	function getOwn(obj,prop) {
		return hasProp(obj, prop) && obj[prop];
	}

	/**
	 * Cycles over properties in an object and calls a function for each
	 * property value. If the function returns a truthy value, then the
	 * iteration is stopped.
	 */
	function eachProp(obj,func) {
		var prop;
		for (prop in obj) {
			if (hasProp(obj, prop)) {
				if (func(obj[prop], prop)) {
					break;
				}
			}
		}
	}

	/**
	 * Simple function to mix in properties from source into target,
	 * but only if target does not already have a property of the same name.
	 */
	function mixin(target,source,force,deepStringMixin) {
		if (source) {
			eachProp(source, function(value,prop) {
				if (force || !hasProp(target, prop)) {
					if (deepStringMixin && typeof value === 'object' && value && !isArray(value) && !isFunction(value) && !(value instanceof RegExp)) {

						if (!target[prop]) {
							target[prop] = {};
						}
						mixin(target[prop], value, force, deepStringMixin);
					} else {
						target[prop] = value;
					}
				}
			});
		}
		return target;
	}

	//Similar to Function.prototype.bind, but the 'this' object is specified
	//first, since it is easier to read/figure out what 'this' will be.
	function bind(obj,fn) {
		return function() {
			return fn.apply(obj, arguments);
		};
	}

	function scripts() {
		return document.getElementsByTagName('script');
	}

	function defaultOnError(err) {
		throw err;
	}

	//Allow getting a global that is expressed in
	//dot notation, like 'a.b.c'.
	function getGlobal(value) {
		if (!value) {
			return value;
		}
		var g = global;
		each(value.split('.'), function(part) {
			g = g[part];
		});
		return g;
	}

	/**
	 * Constructs an error with a pointer to an URL with more information.
	 * @param {String} id the error ID that maps to an ID on a web page.
	 * @param {String} message human readable error.
	 * @param {Error} [err] the original error, if there is one.
	 *
	 * @returns {Error}
	 */
	function makeError(id,msg,err,requireModules) {
		var e = new Error(msg + '\nhttp://requirejs.org/docs/errors.html#' + id);
		e.requireType = id;
		e.requireModules = requireModules;
		if (err) {
			e.originalError = err;
		}
		return e;
	}

	if (typeof define !== 'undefined') {
		//If a define is already in play via another AMD loader,
		//do not overwrite.
		return;
	}

	if (typeof requirejs !== 'undefined') {
		if (isFunction(requirejs)) {
			//Do not overwrite an existing requirejs instance.
			return;
		}
		cfg = requirejs;
		requirejs = undefined;
	}

	//Allow for a require config object
	if (typeof require !== 'undefined' && !isFunction(require)) {
		//assume it is a config object.
		cfg = require;
		require = undefined;
	}

	function newContext(contextName) {
		var inCheckLoaded, Module, context, handlers, checkLoadedTimeoutId, config = {
			//Defaults. Do not set a default for map
			//config to speed up normalize(), which
			//will run faster if there is no default.
			waitSeconds : 7,
			baseUrl : './',
			paths : {},
			bundles : {},
			pkgs : {},
			shim : {},
			config : {}
		}, registry = {},
		//registry of just enabled modules, to speed
		//cycle breaking code when lots of modules
		//are registered, but not activated.
		enabledRegistry = {}, undefEvents = {}, defQueue = [], defined = {}, urlFetched = {}, bundlesMap = {}, requireCounter = 1, unnormalizedCounter = 1;

		/**
		 * Trims the . and .. from an array of path segments.
		 * It will keep a leading path segment if a .. will become
		 * the first path segment, to help with module name lookups,
		 * which act like paths, but can be remapped. But the end result,
		 * all paths that use this function should look normalized.
		 * NOTE: this method MODIFIES the input array.
		 * @param {Array} ary the array of path segments.
		 */
		function trimDots(ary) {
			var i, part;
			for (i = 0; i < ary.length; i++) {
				part = ary[i];
				if (part === '.') {
					ary.splice(i, 1);
					i -= 1;
				} else if (part === '..') {
					// If at the start, or previous value is still ..,
					// keep them so that when converted to a path it may
					// still work when converted to a path, even though
					// as an ID it is less than ideal. In larger point
					// releases, may be better to just kick out an error.
					if (i === 0 || (i == 1 && ary[2] === '..') || ary[i - 1] === '..') {
						continue;
					} else if (i > 0) {
						ary.splice(i - 1, 2);
						i -= 2;
					}
				}
			}
		}

		/**
		 * Given a relative module name, like ./something, normalize it to
		 * a real name that can be mapped to a path.
		 * @param {String} name the relative name
		 * @param {String} baseName a real name that the name arg is relative
		 * to.
		 * @param {Boolean} applyMap apply the map config to the value. Should
		 * only be done if this normalization is for a dependency ID.
		 * @returns {String} normalized name
		 */
		function normalize(name,baseName,applyMap) {
			var pkgMain, mapValue, nameParts, i, j, nameSegment, lastIndex, foundMap, foundI, foundStarMap, starI, normalizedBaseParts, baseParts = (baseName && baseName.split('/')), map = config.map, starMap = map && map['*'];

			//Adjust any relative paths.
			if (name) {
				name = name.split('/');
				lastIndex = name.length - 1;

				// If wanting node ID compatibility, strip .js from end
				// of IDs. Have to do this here, and not in nameToUrl
				// because node allows either .js or non .js to map
				// to same file.
				if (config.nodeIdCompat && jsSuffixRegExp.test(name[lastIndex])) {
					name[lastIndex] = name[lastIndex].replace(jsSuffixRegExp, '');
				}

				// Starts with a '.' so need the baseName
				if (name[0].charAt(0) === '.' && baseParts) {
					//Convert baseName to array, and lop off the last part,
					//so that . matches that 'directory' and not name of the baseName's
					//module. For instance, baseName of 'one/two/three', maps to
					//'one/two/three.js', but we want the directory, 'one/two' for
					//this normalization.
					normalizedBaseParts = baseParts.slice(0, baseParts.length - 1);
					name = normalizedBaseParts.concat(name);
				}

				trimDots(name);
				name = name.join('/');
			}

			//Apply map config if available.
			if (applyMap && map && (baseParts || starMap)) {
				nameParts = name.split('/');

				outerLoop: for (i = nameParts.length; i > 0; i -= 1) {
					nameSegment = nameParts.slice(0, i).join('/');

					if (baseParts) {
						//Find the longest baseName segment match in the config.
						//So, do joins on the biggest to smallest lengths of baseParts.
						for (j = baseParts.length; j > 0; j -= 1) {
							mapValue = getOwn(map, baseParts.slice(0, j).join('/'));

							//baseName segment has config, find if it has one for
							//this name.
							if (mapValue) {
								mapValue = getOwn(mapValue, nameSegment);
								if (mapValue) {
									//Match, update name to the new value.
									foundMap = mapValue;
									foundI = i;
									break outerLoop;
								}
							}
						}
					}

					//Check for a star map match, but just hold on to it,
					//if there is a shorter segment match later in a matching
					//config, then favor over this star map.
					if (!foundStarMap && starMap && getOwn(starMap, nameSegment)) {
						foundStarMap = getOwn(starMap, nameSegment);
						starI = i;
					}
				}

				if (!foundMap && foundStarMap) {
					foundMap = foundStarMap;
					foundI = starI;
				}

				if (foundMap) {
					nameParts.splice(0, foundI, foundMap);
					name = nameParts.join('/');
				}
			}

			// If the name points to a package's name, use
			// the package main instead.
			pkgMain = getOwn(config.pkgs, name);

			return pkgMain ? pkgMain : name;
		}

		function removeScript(name) {
			if (isBrowser) {
				each(scripts(), function(scriptNode) {
					if (scriptNode.getAttribute('data-requiremodule') === name && scriptNode.getAttribute('data-requirecontext') === context.contextName) {
						scriptNode.parentNode.removeChild(scriptNode);
						return true;
					}
				});
			}
		}

		function hasPathFallback(id) {
			var pathConfig = getOwn(config.paths, id);
			if (pathConfig && isArray(pathConfig) && pathConfig.length > 1) {
				//Pop off the first array value, since it failed, and
				//retry
				pathConfig.shift();
				context.require.undef(id);

				//Custom require that does not do map translation, since
				//ID is "absolute", already mapped/resolved.
				context.makeRequire(null, {
					skipMap : true
				})([
					id
				]);

				return true;
			}
		}

		//Turns a plugin!resource to [plugin, resource]
		//with the plugin being undefined if the name
		//did not have a plugin prefix.
		function splitPrefix(name) {
			var prefix, index = name ? name.indexOf('!') : -1;
			if (index > -1) {
				prefix = name.substring(0, index);
				name = name.substring(index + 1, name.length);
			}
			return [
					prefix, name
			];
		}

		/**
		 * Creates a module mapping that includes plugin prefix, module
		 * name, and path. If parentModuleMap is provided it will
		 * also normalize the name via require.normalize()
		 *
		 * @param {String} name the module name
		 * @param {String} [parentModuleMap] parent module map
		 * for the module name, used to resolve relative names.
		 * @param {Boolean} isNormalized: is the ID already normalized.
		 * This is true if this call is done for a define() module ID.
		 * @param {Boolean} applyMap: apply the map config to the ID.
		 * Should only be true if this map is for a dependency.
		 *
		 * @returns {Object}
		 */
		function makeModuleMap(name,parentModuleMap,isNormalized,applyMap) {
			var url, pluginModule, suffix, nameParts, prefix = null, parentName = parentModuleMap ? parentModuleMap.name : null, originalName = name, isDefine = true, normalizedName = '';

			//If no name, then it means it is a require call, generate an
			//internal name.
			if (!name) {
				isDefine = false;
				name = '_@r' + (requireCounter += 1);
			}

			nameParts = splitPrefix(name);
			prefix = nameParts[0];
			name = nameParts[1];

			if (prefix) {
				prefix = normalize(prefix, parentName, applyMap);
				pluginModule = getOwn(defined, prefix);
			}

			//Account for relative paths if there is a base name.
			if (name) {
				if (prefix) {
					if (pluginModule && pluginModule.normalize) {
						//Plugin is loaded, use its normalize method.
						normalizedName = pluginModule.normalize(name, function(name) {
							return normalize(name, parentName, applyMap);
						});
					} else {
						// If nested plugin references, then do not try to
						// normalize, as it will not normalize correctly. This
						// places a restriction on resourceIds, and the longer
						// term solution is not to normalize until plugins are
						// loaded and all normalizations to allow for async
						// loading of a loader plugin. But for now, fixes the
						// common uses. Details in #1131
						normalizedName = name.indexOf('!') === -1 ? normalize(name, parentName, applyMap) : name;
					}
				} else {
					//A regular module.
					normalizedName = normalize(name, parentName, applyMap);

					//Normalized name may be a plugin ID due to map config
					//application in normalize. The map config values must
					//already be normalized, so do not need to redo that part.
					nameParts = splitPrefix(normalizedName);
					prefix = nameParts[0];
					normalizedName = nameParts[1];
					isNormalized = true;

					url = context.nameToUrl(normalizedName);
				}
			}

			//If the id is a plugin id that cannot be determined if it needs
			//normalization, stamp it with a unique ID so two matching relative
			//ids that may conflict can be separate.
			suffix = prefix && !pluginModule && !isNormalized ? '_unnormalized' + (unnormalizedCounter += 1) : '';

			return {
				prefix : prefix,
				name : normalizedName,
				parentMap : parentModuleMap,
				unnormalized : !!suffix,
				url : url,
				originalName : originalName,
				isDefine : isDefine,
				id : (prefix ? prefix + '!' + normalizedName : normalizedName) + suffix
			};
		}

		function getModule(depMap) {
			var id = depMap.id, mod = getOwn(registry, id);

			if (!mod) {
				mod = registry[id] = new context.Module(depMap);
			}

			return mod;
		}

		function on(depMap,name,fn) {
			var id = depMap.id, mod = getOwn(registry, id);

			if (hasProp(defined, id) && (!mod || mod.defineEmitComplete)) {
				if (name === 'defined') {
					fn(defined[id]);
				}
			} else {
				mod = getModule(depMap);
				if (mod.error && name === 'error') {
					fn(mod.error);
				} else {
					mod.on(name, fn);
				}
			}
		}

		function onError(err,errback) {
			var ids = err.requireModules, notified = false;

			if (errback) {
				errback(err);
			} else {
				each(ids, function(id) {
					var mod = getOwn(registry, id);
					if (mod) {
						//Set error on module, so it skips timeout checks.
						mod.error = err;
						if (mod.events.error) {
							notified = true;
							mod.emit('error', err);
						}
					}
				});

				if (!notified) {
					req.onError(err);
				}
			}
		}

		/**
		 * Internal method to transfer globalQueue items to this context's
		 * defQueue.
		 */
		function takeGlobalQueue() {
			//Push all the globalDefQueue items into the context's defQueue
			if (globalDefQueue.length) {
				//Array splice in the values since the context code has a
				//local var ref to defQueue, so cannot just reassign the one
				//on context.
				apsp.apply(defQueue, [
						defQueue.length, 0
				].concat(globalDefQueue));
				globalDefQueue = [];
			}
		}

		handlers = {
			'require' : function(mod) {
				if (mod.require) {
					return mod.require;
				} else {
					return (mod.require = context.makeRequire(mod.map));
				}
			},
			'exports' : function(mod) {
				mod.usingExports = true;
				if (mod.map.isDefine) {
					if (mod.exports) {
						return (defined[mod.map.id] = mod.exports);
					} else {
						return (mod.exports = defined[mod.map.id] = {});
					}
				}
			},
			'module' : function(mod) {
				if (mod.module) {
					return mod.module;
				} else {
					return (mod.module = {
						id : mod.map.id,
						uri : mod.map.url,
						config : function() {
							return getOwn(config.config, mod.map.id) || {};
						},
						exports : mod.exports || (mod.exports = {})
					});
				}
			}
		};

		function cleanRegistry(id) {
			//Clean up machinery used for waiting modules.
			delete registry[id];
			delete enabledRegistry[id];
		}

		function breakCycle(mod,traced,processed) {
			var id = mod.map.id;

			if (mod.error) {
				mod.emit('error', mod.error);
			} else {
				traced[id] = true;
				each(mod.depMaps, function(depMap,i) {
					var depId = depMap.id, dep = getOwn(registry, depId);

					//Only force things that have not completed
					//being defined, so still in the registry,
					//and only if it has not been matched up
					//in the module already.
					if (dep && !mod.depMatched[i] && !processed[depId]) {
						if (getOwn(traced, depId)) {
							mod.defineDep(i, defined[depId]);
							mod.check(); //pass false?
						} else {
							breakCycle(dep, traced, processed);
						}
					}
				});
				processed[id] = true;
			}
		}

		function checkLoaded() {
			var err, usingPathFallback, waitInterval = config.waitSeconds * 1000,
			//It is possible to disable the wait interval by using waitSeconds of 0.
			expired = waitInterval && (context.startTime + waitInterval) < new Date().getTime(), noLoads = [], reqCalls = [], stillLoading = false, needCycleCheck = true;

			//Do not bother if this call was a result of a cycle break.
			if (inCheckLoaded) {
				return;
			}

			inCheckLoaded = true;

			//Figure out the state of all the modules.
			eachProp(enabledRegistry, function(mod) {
				var map = mod.map, modId = map.id;

				//Skip things that are not enabled or in error state.
				if (!mod.enabled) {
					return;
				}

				if (!map.isDefine) {
					reqCalls.push(mod);
				}

				if (!mod.error) {
					//If the module should be executed, and it has not
					//been inited and time is up, remember it.
					if (!mod.inited && expired) {
						if (hasPathFallback(modId)) {
							usingPathFallback = true;
							stillLoading = true;
						} else {
							noLoads.push(modId);
							removeScript(modId);
						}
					} else if (!mod.inited && mod.fetched && map.isDefine) {
						stillLoading = true;
						if (!map.prefix) {
							//No reason to keep looking for unfinished
							//loading. If the only stillLoading is a
							//plugin resource though, keep going,
							//because it may be that a plugin resource
							//is waiting on a non-plugin cycle.
							return (needCycleCheck = false);
						}
					}
				}
			});

			if (expired && noLoads.length) {
				//If wait time expired, throw error of unloaded modules.
				err = makeError('timeout', 'Load timeout for modules: ' + noLoads, null, noLoads);
				err.contextName = context.contextName;
				return onError(err);
			}

			//Not expired, check for a cycle.
			if (needCycleCheck) {
				each(reqCalls, function(mod) {
					breakCycle(mod, {}, {});
				});
			}

			//If still waiting on loads, and the waiting load is something
			//other than a plugin resource, or there are still outstanding
			//scripts, then just try back later.
			if ((!expired || usingPathFallback) && stillLoading) {
				//Something is still waiting to load. Wait for it, but only
				//if a timeout is not already in effect.
				if ((isBrowser || isWebWorker) && !checkLoadedTimeoutId) {
					checkLoadedTimeoutId = setTimeout(function() {
						checkLoadedTimeoutId = 0;
						checkLoaded();
					}, 50);
				}
			}

			inCheckLoaded = false;
		}

		Module = function(map) {
			this.events = getOwn(undefEvents, map.id) || {};
			this.map = map;
			this.shim = getOwn(config.shim, map.id);
			this.depExports = [];
			this.depMaps = [];
			this.depMatched = [];
			this.pluginMaps = {};
			this.depCount = 0;

			/* this.exports this.factory
			   this.depMaps = [],
			   this.enabled, this.fetched
			*/
		};

		Module.prototype = {
			init : function(depMaps,factory,errback,options) {
				options = options || {};

				//Do not do more inits if already done. Can happen if there
				//are multiple define calls for the same module. That is not
				//a normal, common case, but it is also not unexpected.
				if (this.inited) {
					return;
				}

				this.factory = factory;

				if (errback) {
					//Register for errors on this module.
					this.on('error', errback);
				} else if (this.events.error) {
					//If no errback already, but there are error listeners
					//on this module, set up an errback to pass to the deps.
					errback = bind(this, function(err) {
						this.emit('error', err);
					});
				}

				//Do a copy of the dependency array, so that
				//source inputs are not modified. For example
				//"shim" deps are passed in here directly, and
				//doing a direct modification of the depMaps array
				//would affect that config.
				this.depMaps = depMaps && depMaps.slice(0);

				this.errback = errback;

				//Indicate this module has be initialized
				this.inited = true;

				this.ignore = options.ignore;

				//Could have option to init this module in enabled mode,
				//or could have been previously marked as enabled. However,
				//the dependencies are not known until init is called. So
				//if enabled previously, now trigger dependencies as enabled.
				if (options.enabled || this.enabled) {
					//Enable this module and dependencies.
					//Will call this.check()
					this.enable();
				} else {
					this.check();
				}
			},

			defineDep : function(i,depExports) {
				//Because of cycles, defined callback for a given
				//export can be called more than once.
				if (!this.depMatched[i]) {
					this.depMatched[i] = true;
					this.depCount -= 1;
					this.depExports[i] = depExports;
				}
			},

			fetch : function() {
				if (this.fetched) {
					return;
				}
				this.fetched = true;

				context.startTime = (new Date()).getTime();

				var map = this.map;

				//If the manager is for a plugin managed resource,
				//ask the plugin to load it now.
				if (this.shim) {
					context.makeRequire(this.map, {
						enableBuildCallback : true
					})(this.shim.deps || [], bind(this, function() {
						return map.prefix ? this.callPlugin() : this.load();
					}));
				} else {
					//Regular dependency.
					return map.prefix ? this.callPlugin() : this.load();
				}
			},

			load : function() {
				var url = this.map.url;

				//Regular dependency.
				if (!urlFetched[url]) {
					urlFetched[url] = true;
					context.load(this.map.id, url);
				}
			},

			/**
			 * Checks if the module is ready to define itself, and if so,
			 * define it.
			 */
			check : function() {
				if (!this.enabled || this.enabling) {
					return;
				}

				var err, cjsModule, id = this.map.id, depExports = this.depExports, exports = this.exports, factory = this.factory;

				if (!this.inited) {
					this.fetch();
				} else if (this.error) {
					this.emit('error', this.error);
				} else if (!this.defining) {
					//The factory could trigger another require call
					//that would result in checking this module to
					//define itself again. If already in the process
					//of doing that, skip this work.
					this.defining = true;

					if (this.depCount < 1 && !this.defined) {
						if (isFunction(factory)) {
							//If there is an error listener, favor passing
							//to that instead of throwing an error. However,
							//only do it for define()'d  modules. require
							//errbacks should not be called for failures in
							//their callbacks (#699). However if a global
							//onError is set, use that.
							if ((this.events.error && this.map.isDefine) || req.onError !== defaultOnError) {
								try {
									exports = context.execCb(id, factory, depExports, exports);
								} catch (e) {
									err = e;
								}
							} else {
								exports = context.execCb(id, factory, depExports, exports);
							}

							// Favor return value over exports. If node/cjs in play,
							// then will not have a return value anyway. Favor
							// module.exports assignment over exports object.
							if (this.map.isDefine && exports === undefined) {
								cjsModule = this.module;
								if (cjsModule) {
									exports = cjsModule.exports;
								} else if (this.usingExports) {
									//exports already set the defined value.
									exports = this.exports;
								}
							}

							if (err) {
								err.requireMap = this.map;
								err.requireModules = this.map.isDefine ? [
									this.map.id
								] : null;
								err.requireType = this.map.isDefine ? 'define' : 'require';
								return onError((this.error = err));
							}

						} else {
							//Just a literal value
							exports = factory;
						}

						this.exports = exports;

						if (this.map.isDefine && !this.ignore) {
							defined[id] = exports;

							if (req.onResourceLoad) {
								req.onResourceLoad(context, this.map, this.depMaps);
							}
						}

						//Clean up
						cleanRegistry(id);

						this.defined = true;
					}

					//Finished the define stage. Allow calling check again
					//to allow define notifications below in the case of a
					//cycle.
					this.defining = false;

					if (this.defined && !this.defineEmitted) {
						this.defineEmitted = true;
						this.emit('defined', this.exports);
						this.defineEmitComplete = true;
					}

				}
			},

			callPlugin : function() {
				var map = this.map, id = map.id,
				//Map already normalized the prefix.
				pluginMap = makeModuleMap(map.prefix);

				//Mark this as a dependency for this plugin, so it
				//can be traced for cycles.
				this.depMaps.push(pluginMap);

				on(pluginMap, 'defined', bind(this, function(plugin) {
					var load, normalizedMap, normalizedMod, bundleId = getOwn(bundlesMap, this.map.id), name = this.map.name, parentName = this.map.parentMap ? this.map.parentMap.name : null, localRequire = context.makeRequire(map.parentMap, {
						enableBuildCallback : true
					});

					//If current map is not normalized, wait for that
					//normalized name to load instead of continuing.
					if (this.map.unnormalized) {
						//Normalize the ID if the plugin allows it.
						if (plugin.normalize) {
							name = plugin.normalize(name, function(name) {
								return normalize(name, parentName, true);
							}) || '';
						}

						//prefix and name should already be normalized, no need
						//for applying map config again either.
						normalizedMap = makeModuleMap(map.prefix + '!' + name, this.map.parentMap);
						on(normalizedMap, 'defined', bind(this, function(value) {
							this.init([], function() {
								return value;
							}, null, {
								enabled : true,
								ignore : true
							});
						}));

						normalizedMod = getOwn(registry, normalizedMap.id);
						if (normalizedMod) {
							//Mark this as a dependency for this plugin, so it
							//can be traced for cycles.
							this.depMaps.push(normalizedMap);

							if (this.events.error) {
								normalizedMod.on('error', bind(this, function(err) {
									this.emit('error', err);
								}));
							}
							normalizedMod.enable();
						}

						return;
					}

					//If a paths config, then just load that file instead to
					//resolve the plugin, as it is built into that paths layer.
					if (bundleId) {
						this.map.url = context.nameToUrl(bundleId);
						this.load();
						return;
					}

					load = bind(this, function(value) {
						this.init([], function() {
							return value;
						}, null, {
							enabled : true
						});
					});

					load.error = bind(this, function(err) {
						this.inited = true;
						this.error = err;
						err.requireModules = [
							id
						];

						//Remove temp unnormalized modules for this module,
						//since they will never be resolved otherwise now.
						eachProp(registry, function(mod) {
							if (mod.map.id.indexOf(id + '_unnormalized') === 0) {
								cleanRegistry(mod.map.id);
							}
						});

						onError(err);
					});

					//Allow plugins to load other code without having to know the
					//context or how to 'complete' the load.
					load.fromText = bind(this, function(text,textAlt) {
						/*jslint evil: true */
						var moduleName = map.name, moduleMap = makeModuleMap(moduleName), hasInteractive = useInteractive;

						//As of 2.1.0, support just passing the text, to reinforce
						//fromText only being called once per resource. Still
						//support old style of passing moduleName but discard
						//that moduleName in favor of the internal ref.
						if (textAlt) {
							text = textAlt;
						}

						//Turn off interactive script matching for IE for any define
						//calls in the text, then turn it back on at the end.
						if (hasInteractive) {
							useInteractive = false;
						}

						//Prime the system by creating a module instance for
						//it.
						getModule(moduleMap);

						//Transfer any config to this other module.
						if (hasProp(config.config, id)) {
							config.config[moduleName] = config.config[id];
						}

						try {
							req.exec(text);
						} catch (e) {
							return onError(makeError('fromtexteval', 'fromText eval for ' + id + ' failed: ' + e, e, [
								id
							]));
						}

						if (hasInteractive) {
							useInteractive = true;
						}

						//Mark this as a dependency for the plugin
						//resource
						this.depMaps.push(moduleMap);

						//Support anonymous modules.
						context.completeLoad(moduleName);

						//Bind the value of that module to the value for this
						//resource ID.
						localRequire([
							moduleName
						], load);
					});

					//Use parentName here since the plugin's name is not reliable,
					//could be some weird string with no path that actually wants to
					//reference the parentName's path.
					plugin.load(map.name, localRequire, load, config);
				}));

				context.enable(pluginMap, this);
				this.pluginMaps[pluginMap.id] = pluginMap;
			},

			enable : function() {
				enabledRegistry[this.map.id] = this;
				this.enabled = true;

				//Set flag mentioning that the module is enabling,
				//so that immediate calls to the defined callbacks
				//for dependencies do not trigger inadvertent load
				//with the depCount still being zero.
				this.enabling = true;

				//Enable each dependency
				each(this.depMaps, bind(this, function(depMap,i) {
					var id, mod, handler;

					if (typeof depMap === 'string') {
						//Dependency needs to be converted to a depMap
						//and wired up to this module.
						depMap = makeModuleMap(depMap, (this.map.isDefine ? this.map : this.map.parentMap), false, !this.skipMap);
						this.depMaps[i] = depMap;

						handler = getOwn(handlers, depMap.id);

						if (handler) {
							this.depExports[i] = handler(this);
							return;
						}

						this.depCount += 1;

						on(depMap, 'defined', bind(this, function(depExports) {
							this.defineDep(i, depExports);
							this.check();
						}));

						if (this.errback) {
							on(depMap, 'error', bind(this, this.errback));
						}
					}

					id = depMap.id;
					mod = registry[id];

					//Skip special modules like 'require', 'exports', 'module'
					//Also, don't call enable if it is already enabled,
					//important in circular dependency cases.
					if (!hasProp(handlers, id) && mod && !mod.enabled) {
						context.enable(depMap, this);
					}
				}));

				//Enable each plugin that is used in
				//a dependency
				eachProp(this.pluginMaps, bind(this, function(pluginMap) {
					var mod = getOwn(registry, pluginMap.id);
					if (mod && !mod.enabled) {
						context.enable(pluginMap, this);
					}
				}));

				this.enabling = false;

				this.check();
			},

			on : function(name,cb) {
				var cbs = this.events[name];
				if (!cbs) {
					cbs = this.events[name] = [];
				}
				cbs.push(cb);
			},

			emit : function(name,evt) {
				each(this.events[name], function(cb) {
					cb(evt);
				});
				if (name === 'error') {
					//Now that the error handler was triggered, remove
					//the listeners, since this broken Module instance
					//can stay around for a while in the registry.
					delete this.events[name];
				}
			}
		};

		function callGetModule(args) {
			//Skip modules already defined.
			if (!hasProp(defined, args[0])) {
				getModule(makeModuleMap(args[0], null, true)).init(args[1], args[2]);
			}
		}

		function removeListener(node,func,name,ieName) {
			//Favor detachEvent because of IE9
			//issue, see attachEvent/addEventListener comment elsewhere
			//in this file.
			if (node.detachEvent && !isOpera) {
				//Probably IE. If not it will throw an error, which will be
				//useful to know.
				if (ieName) {
					node.detachEvent(ieName, func);
				}
			} else {
				node.removeEventListener(name, func, false);
			}
		}

		/**
		 * Given an event from a script node, get the requirejs info from it,
		 * and then removes the event listeners on the node.
		 * @param {Event} evt
		 * @returns {Object}
		 */
		function getScriptData(evt) {
			//Using currentTarget instead of target for Firefox 2.0's sake. Not
			//all old browsers will be supported, but this one was easy enough
			//to support and still makes sense.
			var node = evt.currentTarget || evt.srcElement;

			//Remove the listeners once here.
			removeListener(node, context.onScriptLoad, 'load', 'onreadystatechange');
			removeListener(node, context.onScriptError, 'error');

			return {
				node : node,
				id : node && node.getAttribute('data-requiremodule')
			};
		}

		function intakeDefines() {
			var args;

			//Any defined modules in the global queue, intake them now.
			takeGlobalQueue();

			//Make sure any remaining defQueue items get properly processed.
			while (defQueue.length) {
				args = defQueue.shift();
				if (args[0] === null) {
					return onError(makeError('mismatch', 'Mismatched anonymous define() module: ' + args[args.length - 1]));
				} else {
					//args are id, deps, factory. Should be normalized by the
					//define() function.
					callGetModule(args);
				}
			}
		}

		context = {
			config : config,
			contextName : contextName,
			registry : registry,
			defined : defined,
			urlFetched : urlFetched,
			defQueue : defQueue,
			Module : Module,
			makeModuleMap : makeModuleMap,
			nextTick : req.nextTick,
			onError : onError,

			/**
			 * Set a configuration for the context.
			 * @param {Object} cfg config object to integrate.
			 */
			configure : function(cfg) {
				//Make sure the baseUrl ends in a slash.
				if (cfg.baseUrl) {
					if (cfg.baseUrl.charAt(cfg.baseUrl.length - 1) !== '/') {
						cfg.baseUrl += '/';
					}
				}

				//Save off the paths since they require special processing,
				//they are additive.
				var shim = config.shim, objs = {
					paths : true,
					bundles : true,
					config : true,
					map : true
				};

				eachProp(cfg, function(value,prop) {
					if (objs[prop]) {
						if (!config[prop]) {
							config[prop] = {};
						}
						mixin(config[prop], value, true, true);
					} else {
						config[prop] = value;
					}
				});

				//Reverse map the bundles
				if (cfg.bundles) {
					eachProp(cfg.bundles, function(value,prop) {
						each(value, function(v) {
							if (v !== prop) {
								bundlesMap[v] = prop;
							}
						});
					});
				}

				//Merge shim
				if (cfg.shim) {
					eachProp(cfg.shim, function(value,id) {
						//Normalize the structure
						if (isArray(value)) {
							value = {
								deps : value
							};
						}
						if ((value.exports || value.init) && !value.exportsFn) {
							value.exportsFn = context.makeShimExports(value);
						}
						shim[id] = value;
					});
					config.shim = shim;
				}

				//Adjust packages if necessary.
				if (cfg.packages) {
					each(cfg.packages, function(pkgObj) {
						var location, name;

						pkgObj = typeof pkgObj === 'string' ? {
							name : pkgObj
						} : pkgObj;

						name = pkgObj.name;
						location = pkgObj.location;
						if (location) {
							config.paths[name] = pkgObj.location;
						}

						//Save pointer to main module ID for pkg name.
						//Remove leading dot in main, so main paths are normalized,
						//and remove any trailing .js, since different package
						//envs have different conventions: some use a module name,
						//some use a file name.
						config.pkgs[name] = pkgObj.name + '/' + (pkgObj.main || 'main').replace(currDirRegExp, '').replace(jsSuffixRegExp, '');
					});
				}

				//If there are any "waiting to execute" modules in the registry,
				//update the maps for them, since their info, like URLs to load,
				//may have changed.
				eachProp(registry, function(mod,id) {
					//If module already has init called, since it is too
					//late to modify them, and ignore unnormalized ones
					//since they are transient.
					if (!mod.inited && !mod.map.unnormalized) {
						mod.map = makeModuleMap(id);
					}
				});

				//If a deps array or a config callback is specified, then call
				//require with those args. This is useful when require is defined as a
				//config object before require.js is loaded.
				if (cfg.deps || cfg.callback) {
					context.require(cfg.deps || [], cfg.callback);
				}
			},

			makeShimExports : function(value) {
				function fn() {
					var ret;
					if (value.init) {
						ret = value.init.apply(global, arguments);
					}
					return ret || (value.exports && getGlobal(value.exports));
				}
				return fn;
			},

			makeRequire : function(relMap,options) {
				options = options || {};

				function localRequire(deps,callback,errback) {
					var id, map, requireMod;

					if (options.enableBuildCallback && callback && isFunction(callback)) {
						callback.__requireJsBuild = true;
					}

					if (typeof deps === 'string') {
						if (isFunction(callback)) {
							//Invalid call
							return onError(makeError('requireargs', 'Invalid require call'), errback);
						}

						//If require|exports|module are requested, get the
						//value for them from the special handlers. Caveat:
						//this only works while module is being defined.
						if (relMap && hasProp(handlers, deps)) {
							return handlers[deps](registry[relMap.id]);
						}

						//Synchronous access to one module. If require.get is
						//available (as in the Node adapter), prefer that.
						if (req.get) {
							return req.get(context, deps, relMap, localRequire);
						}

						//Normalize module name, if it contains . or ..
						map = makeModuleMap(deps, relMap, false, true);
						id = map.id;

						if (!hasProp(defined, id)) {
							return onError(makeError('notloaded', 'Module name "' + id + '" has not been loaded yet for context: ' + contextName + (relMap ? '' : '. Use require([])')));
						}
						return defined[id];
					}

					//Grab defines waiting in the global queue.
					intakeDefines();

					//Mark all the dependencies as needing to be loaded.
					context.nextTick(function() {
						//Some defines could have been added since the
						//require call, collect them.
						intakeDefines();

						requireMod = getModule(makeModuleMap(null, relMap));

						//Store if map config should be applied to this require
						//call for dependencies.
						requireMod.skipMap = options.skipMap;

						requireMod.init(deps, callback, errback, {
							enabled : true
						});

						checkLoaded();
					});

					return localRequire;
				}

				mixin(localRequire, {
					isBrowser : isBrowser,

					/**
					 * Converts a module name + .extension into an URL path.
					 * *Requires* the use of a module name. It does not support using
					 * plain URLs like nameToUrl.
					 */
					toUrl : function(moduleNamePlusExt) {
						var ext, index = moduleNamePlusExt.lastIndexOf('.'), segment = moduleNamePlusExt.split('/')[0], isRelative = segment === '.' || segment === '..';

						//Have a file extension alias, and it is not the
						//dots from a relative path.
						if (index !== -1 && (!isRelative || index > 1)) {
							ext = moduleNamePlusExt.substring(index, moduleNamePlusExt.length);
							moduleNamePlusExt = moduleNamePlusExt.substring(0, index);
						}

						return context.nameToUrl(normalize(moduleNamePlusExt, relMap && relMap.id, true), ext, true);
					},

					defined : function(id) {
						return hasProp(defined, makeModuleMap(id, relMap, false, true).id);
					},

					specified : function(id) {
						id = makeModuleMap(id, relMap, false, true).id;
						return hasProp(defined, id) || hasProp(registry, id);
					}
				});

				//Only allow undef on top level require calls
				if (!relMap) {
					localRequire.undef = function(id) {
						//Bind any waiting define() calls to this context,
						//fix for #408
						takeGlobalQueue();

						var map = makeModuleMap(id, relMap, true), mod = getOwn(registry, id);

						removeScript(id);

						delete defined[id];
						delete urlFetched[map.url];
						delete undefEvents[id];

						//Clean queued defines too. Go backwards
						//in array so that the splices do not
						//mess up the iteration.
						eachReverse(defQueue, function(args,i) {
							if (args[0] === id) {
								defQueue.splice(i, 1);
							}
						});

						if (mod) {
							//Hold on to listeners in case the
							//module will be attempted to be reloaded
							//using a different config.
							if (mod.events.defined) {
								undefEvents[id] = mod.events;
							}

							cleanRegistry(id);
						}
					};
				}

				return localRequire;
			},

			/**
			 * Called to enable a module if it is still in the registry
			 * awaiting enablement. A second arg, parent, the parent module,
			 * is passed in for context, when this method is overridden by
			 * the optimizer. Not shown here to keep code compact.
			 */
			enable : function(depMap) {
				var mod = getOwn(registry, depMap.id);
				if (mod) {
					getModule(depMap).enable();
				}
			},

			/**
			 * Internal method used by environment adapters to complete a load event.
			 * A load event could be a script load or just a load pass from a synchronous
			 * load call.
			 * @param {String} moduleName the name of the module to potentially complete.
			 */
			completeLoad : function(moduleName) {
				var found, args, mod, shim = getOwn(config.shim, moduleName) || {}, shExports = shim.exports;

				takeGlobalQueue();

				while (defQueue.length) {
					args = defQueue.shift();
					if (args[0] === null) {
						args[0] = moduleName;
						//If already found an anonymous module and bound it
						//to this name, then this is some other anon module
						//waiting for its completeLoad to fire.
						if (found) {
							break;
						}
						found = true;
					} else if (args[0] === moduleName) {
						//Found matching define call for this script!
						found = true;
					}

					callGetModule(args);
				}

				//Do this after the cycle of callGetModule in case the result
				//of those calls/init calls changes the registry.
				mod = getOwn(registry, moduleName);

				if (!found && !hasProp(defined, moduleName) && mod && !mod.inited) {
					if (config.enforceDefine && (!shExports || !getGlobal(shExports))) {
						if (hasPathFallback(moduleName)) {
							return;
						} else {
							return onError(makeError('nodefine', 'No define call for ' + moduleName, null, [
								moduleName
							]));
						}
					} else {
						//A script that does not call define(), so just simulate
						//the call for it.
						callGetModule([
								moduleName, (shim.deps || []), shim.exportsFn
						]);
					}
				}

				checkLoaded();
			},

			/**
			 * Converts a module name to a file path. Supports cases where
			 * moduleName may actually be just an URL.
			 * Note that it **does not** call normalize on the moduleName,
			 * it is assumed to have already been normalized. This is an
			 * internal API, not a public one. Use toUrl for the public API.
			 */
			nameToUrl : function(moduleName,ext,skipExt) {
				var paths, syms, i, parentModule, url, parentPath, bundleId, pkgMain = getOwn(config.pkgs, moduleName);

				if (pkgMain) {
					moduleName = pkgMain;
				}

				bundleId = getOwn(bundlesMap, moduleName);

				if (bundleId) {
					return context.nameToUrl(bundleId, ext, skipExt);
				}

				//If a colon is in the URL, it indicates a protocol is used and it is just
				//an URL to a file, or if it starts with a slash, contains a query arg (i.e. ?)
				//or ends with .js, then assume the user meant to use an url and not a module id.
				//The slash is important for protocol-less URLs as well as full paths.
				if (req.jsExtRegExp.test(moduleName)) {
					//Just a plain path, not module name lookup, so just return it.
					//Add extension if it is included. This is a bit wonky, only non-.js things pass
					//an extension, this method probably needs to be reworked.
					url = moduleName + (ext || '');
				} else {
					//A module that needs to be converted to a path.
					paths = config.paths;

					syms = moduleName.split('/');
					//For each module name segment, see if there is a path
					//registered for it. Start with most specific name
					//and work up from it.
					for (i = syms.length; i > 0; i -= 1) {
						parentModule = syms.slice(0, i).join('/');

						parentPath = getOwn(paths, parentModule);
						if (parentPath) {
							//If an array, it means there are a few choices,
							//Choose the one that is desired
							if (isArray(parentPath)) {
								parentPath = parentPath[0];
							}
							syms.splice(0, i, parentPath);
							break;
						}
					}

					//Join the path parts together, then figure out if baseUrl is needed.
					url = syms.join('/');
					url += (ext || (/^data\:|\?/.test(url) || skipExt ? '' : '.js'));
					url = (url.charAt(0) === '/' || url.match(/^[\w\+\.\-]+:/) ? '' : config.baseUrl) + url;
				}

				return config.urlArgs ? url + ((url.indexOf('?') === -1 ? '?' : '&') + config.urlArgs) : url;
			},

			//Delegates to req.load. Broken out as a separate function to
			//allow overriding in the optimizer.
			load : function(id,url) {
				req.load(context, id, url);
			},

			/**
			 * Executes a module callback function. Broken out as a separate function
			 * solely to allow the build system to sequence the files in the built
			 * layer in the right sequence.
			 *
			 * @private
			 */
			execCb : function(name,callback,args,exports) {
				return callback.apply(exports, args);
			},

			/**
			 * callback for script loads, used to check status of loading.
			 *
			 * @param {Event} evt the event from the browser for the script
			 * that was loaded.
			 */
			onScriptLoad : function(evt) {
				//Using currentTarget instead of target for Firefox 2.0's sake. Not
				//all old browsers will be supported, but this one was easy enough
				//to support and still makes sense.
				if (evt.type === 'load' || (readyRegExp.test((evt.currentTarget || evt.srcElement).readyState))) {
					//Reset interactive script so a script node is not held onto for
					//to long.
					interactiveScript = null;

					//Pull out the name of the module and the context.
					var data = getScriptData(evt);
					context.completeLoad(data.id);
				}
			},

			/**
			 * Callback for script errors.
			 */
			onScriptError : function(evt) {
				var data = getScriptData(evt);
				if (!hasPathFallback(data.id)) {
					return onError(makeError('scripterror', 'Script error for: ' + data.id, evt, [
						data.id
					]));
				}
			}
		};

		context.require = context.makeRequire();
		return context;
	}

	/**
	 * Main entry point.
	 *
	 * If the only argument to require is a string, then the module that
	 * is represented by that string is fetched for the appropriate context.
	 *
	 * If the first argument is an array, then it will be treated as an array
	 * of dependency string names to fetch. An optional function callback can
	 * be specified to execute when all of those dependencies are available.
	 *
	 * Make a local req variable to help Caja compliance (it assumes things
	 * on a require that are not standardized), and to give a short
	 * name for minification/local scope use.
	 */
	req = requirejs = function(deps,callback,errback,optional) {

		//Find the right context, use default
		var context, config, contextName = defContextName;

		// Determine if have config object in the call.
		if (!isArray(deps) && typeof deps !== 'string') {
			// deps is a config object
			config = deps;
			if (isArray(callback)) {
				// Adjust args if there are dependencies
				deps = callback;
				callback = errback;
				errback = optional;
			} else {
				deps = [];
			}
		}

		if (config && config.context) {
			contextName = config.context;
		}

		context = getOwn(contexts, contextName);
		if (!context) {
			context = contexts[contextName] = req.s.newContext(contextName);
		}

		if (config) {
			context.configure(config);
		}

		return context.require(deps, callback, errback);
	};

	/**
	 * Support require.config() to make it easier to cooperate with other
	 * AMD loaders on globally agreed names.
	 */
	req.config = function(config) {
		return req(config);
	};

	/**
	 * Execute something after the current tick
	 * of the event loop. Override for other envs
	 * that have a better solution than setTimeout.
	 * @param  {Function} fn function to execute later.
	 */
	req.nextTick = typeof setTimeout !== 'undefined' ? function(fn) {
		setTimeout(fn, 4);
	} : function(fn) {
		fn();
	};

	/**
	 * Export require as a global, but only if it does not already exist.
	 */
	if (!require) {
		require = req;
	}

	req.version = version;

	//Used to filter out dependencies that are already paths.
	req.jsExtRegExp = /^\/|:|\?|\.js$/;
	req.isBrowser = isBrowser;
	s = req.s = {
		contexts : contexts,
		newContext : newContext
	};

	//Create default context.
	req({});

	//Exports some context-sensitive methods on global require.
	each([
			'toUrl', 'undef', 'defined', 'specified'
	], function(prop) {
		//Reference from contexts instead of early binding to default context,
		//so that during builds, the latest instance of the default context
		//with its config gets used.
		req[prop] = function() {
			var ctx = contexts[defContextName];
			return ctx.require[prop].apply(ctx, arguments);
		};
	});

	if (isBrowser) {
		head = s.head = document.getElementsByTagName('head')[0];
		//If BASE tag is in play, using appendChild is a problem for IE6.
		//When that browser dies, this can be removed. Details in this jQuery bug:
		//http://dev.jquery.com/ticket/2709
		baseElement = document.getElementsByTagName('base')[0];
		if (baseElement) {
			head = s.head = baseElement.parentNode;
		}
	}

	/**
	 * Any errors that require explicitly generates will be passed to this
	 * function. Intercept/override it if you want custom error handling.
	 * @param {Error} err the error object.
	 */
	req.onError = defaultOnError;

	/**
	 * Creates the node for the load command. Only used in browser envs.
	 */
	req.createNode = function(config,moduleName,url) {
		var node = config.xhtml ? document.createElementNS('http://www.w3.org/1999/xhtml', 'html:script') : document.createElement('script');
		node.type = config.scriptType || 'text/javascript';
		node.charset = 'utf-8';
		node.async = true;
		return node;
	};

	/**
	 * Does the request to load a module for the browser case.
	 * Make this a separate function to allow other environments
	 * to override it.
	 *
	 * @param {Object} context the require context to find state.
	 * @param {String} moduleName the name of the module.
	 * @param {Object} url the URL to the module.
	 */
	req.load = function(context,moduleName,url) {
		var config = (context && context.config) || {}, node;
		if (isBrowser) {
			//In the browser so use a script tag
			node = req.createNode(config, moduleName, url);

			node.setAttribute('data-requirecontext', context.contextName);
			node.setAttribute('data-requiremodule', moduleName);

			//Set up load listener. Test attachEvent first because IE9 has
			//a subtle issue in its addEventListener and script onload firings
			//that do not match the behavior of all other browsers with
			//addEventListener support, which fire the onload event for a
			//script right after the script execution. See:
			//https://connect.microsoft.com/IE/feedback/details/648057/script-onload-event-is-not-fired-immediately-after-script-execution
			//UNFORTUNATELY Opera implements attachEvent but does not follow the script
			//script execution mode.
			if (node.attachEvent &&
			//Check if node.attachEvent is artificially added by custom script or
			//natively supported by browser
			//read https://github.com/jrburke/requirejs/issues/187
			//if we can NOT find [native code] then it must NOT natively supported.
			//in IE8, node.attachEvent does not have toString()
			//Note the test for "[native code" with no closing brace, see:
			//https://github.com/jrburke/requirejs/issues/273
			!(node.attachEvent.toString && node.attachEvent.toString().indexOf('[native code') < 0) && !isOpera) {
				//Probably IE. IE (at least 6-8) do not fire
				//script onload right after executing the script, so
				//we cannot tie the anonymous define call to a name.
				//However, IE reports the script as being in 'interactive'
				//readyState at the time of the define call.
				useInteractive = true;

				node.attachEvent('onreadystatechange', context.onScriptLoad);
				//It would be great to add an error handler here to catch
				//404s in IE9+. However, onreadystatechange will fire before
				//the error handler, so that does not help. If addEventListener
				//is used, then IE will fire error before load, but we cannot
				//use that pathway given the connect.microsoft.com issue
				//mentioned above about not doing the 'script execute,
				//then fire the script load event listener before execute
				//next script' that other browsers do.
				//Best hope: IE10 fixes the issues,
				//and then destroys all installs of IE 6-9.
				//node.attachEvent('onerror', context.onScriptError);
			} else {
				node.addEventListener('load', context.onScriptLoad, false);
				node.addEventListener('error', context.onScriptError, false);
			}
			node.src = url;

			//For some cache cases in IE 6-8, the script executes before the end
			//of the appendChild execution, so to tie an anonymous define
			//call to the module name (which is stored on the node), hold on
			//to a reference to this node, but clear after the DOM insertion.
			currentlyAddingScript = node;
			if (baseElement) {
				head.insertBefore(node, baseElement);
			} else {
				head.appendChild(node);
			}
			currentlyAddingScript = null;

			return node;
		} else if (isWebWorker) {
			try {
				//In a web worker, use importScripts. This is not a very
				//efficient use of importScripts, importScripts will block until
				//its script is downloaded and evaluated. However, if web workers
				//are in play, the expectation that a build has been done so that
				//only one script needs to be loaded anyway. This may need to be
				//reevaluated if other use cases become common.
				importScripts(url);

				//Account for anonymous modules
				context.completeLoad(moduleName);
			} catch (e) {
				context.onError(makeError('importscripts', 'importScripts failed for ' + moduleName + ' at ' + url, e, [
					moduleName
				]));
			}
		}
	};

	function getInteractiveScript() {
		if (interactiveScript && interactiveScript.readyState === 'interactive') {
			return interactiveScript;
		}

		eachReverse(scripts(), function(script) {
			if (script.readyState === 'interactive') {
				return (interactiveScript = script);
			}
		});
		return interactiveScript;
	}

	//Look for a data-main script attribute, which could also adjust the baseUrl.
	if (isBrowser && !cfg.skipDataMain) {
		//Figure out baseUrl. Get it from the script tag with require.js in it.
		eachReverse(scripts(), function(script) {
			//Set the 'head' where we can append children by
			//using the script's parent.
			if (!head) {
				head = script.parentNode;
			}

			//Look for a data-main attribute to set main script for the page
			//to load. If it is there, the path to data main becomes the
			//baseUrl, if it is not already set.
			dataMain = script.getAttribute('data-main');
			if (dataMain) {
				//Preserve dataMain in case it is a path (i.e. contains '?')
				mainScript = dataMain;

				//Set final baseUrl if there is not already an explicit one.
				if (!cfg.baseUrl) {
					//Pull off the directory of data-main for use as the
					//baseUrl.
					src = mainScript.split('/');
					mainScript = src.pop();
					subPath = src.length ? src.join('/') + '/' : './';

					cfg.baseUrl = subPath;
				}

				//Strip off any trailing .js since mainScript is now
				//like a module name.
				mainScript = mainScript.replace(jsSuffixRegExp, '');

				//If mainScript is still a path, fall back to dataMain
				if (req.jsExtRegExp.test(mainScript)) {
					mainScript = dataMain;
				}

				//Put the data-main script in the files to load.
				cfg.deps = cfg.deps ? cfg.deps.concat(mainScript) : [
					mainScript
				];

				return true;
			}
		});
	}

	/**
	 * The function that handles definitions of modules. Differs from
	 * require() in that a string for the module should be the first argument,
	 * and the function to execute after dependencies are loaded should
	 * return a value to define the module corresponding to the first argument's
	 * name.
	 */
	define = function(name,deps,callback) {
		var node, context;

		//Allow for anonymous modules
		if (typeof name !== 'string') {
			//Adjust args appropriately
			callback = deps;
			deps = name;
			name = null;
		}

		//This module may not have dependencies
		if (!isArray(deps)) {
			callback = deps;
			deps = null;
		}

		//If no name, and callback is a function, then figure out if it a
		//CommonJS thing with dependencies.
		if (!deps && isFunction(callback)) {
			deps = [];
			//Remove comments from the callback string,
			//look for require calls, and pull them into the dependencies,
			//but only if there are function args.
			if (callback.length) {
				callback.toString().replace(commentRegExp, '').replace(cjsRequireRegExp, function(match,dep) {
					deps.push(dep);
				});

				//May be a CommonJS thing even without require calls, but still
				//could use exports, and module. Avoid doing exports and module
				//work though if it just needs require.
				//REQUIRES the function to expect the CommonJS variables in the
				//order listed below.
				deps = (callback.length === 1 ? [
					'require'
				] : [
						'require', 'exports', 'module'
				]).concat(deps);
			}
		}

		//If in IE 6-8 and hit an anonymous define() call, do the interactive
		//work.
		if (useInteractive) {
			node = currentlyAddingScript || getInteractiveScript();
			if (node) {
				if (!name) {
					name = node.getAttribute('data-requiremodule');
				}
				context = contexts[node.getAttribute('data-requirecontext')];
			}
		}

		//Always save off evaluating the def call until the script onload handler.
		//This allows multiple modules to be in a file without prematurely
		//tracing dependencies, and allows for anonymous module support,
		//where the module name is not known until the script onload event
		//occurs. If no context, use the global queue, and get it processed
		//in the onscript load callback.
		(context ? context.defQueue : globalDefQueue).push([
				name, deps, callback
		]);
	};

	define.amd = {
		jQuery : true
	};

	/**
	 * Executes the text. Normally just uses eval, but can be modified
	 * to use a better, environment-specific call. Only used for transpiling
	 * loader plugins, not for plain JS modules.
	 * @param {String} text the text to execute/evaluate.
	 */
	req.exec = function(text) {
		/*jslint evil: true */
		return eval(text);
	};

	//Set up with config info.
	req(cfg);
}(this));
```

  

Documentation generated by JSDoc 3.4.2 on Sat Oct 08 2016 21:42:26 GMT+0100 (BST) using the docdash theme.
